# Comparison of the gene expression profiles of endometrial and trophoblastic cells in women with recurrent miscarriage: A bioinformatics approach

**DOI:** 10.18502/ijrm.v22i6.16800

**Published:** 2024-08-05

**Authors:** Kambiz Ahmadi, Somayeh Reiisi, Zahra Habibi

**Affiliations:** ^1^Department of Computer Sciences, Faculty of Mathematical Sciences, Shahrekord University, Shahrekord, Iran.; ^2^Department of Genetics, Faculty of Basic Sciences, Shahrekord University, Shahrekord, Iran.; ^3^Department of Women and Family Affairs, Chaharmahal and Bakhtiari Governorate, Shahrekord, Iran.

**Keywords:** Recurrent miscarriage, Transcriptome profile, Gene ontology, Bioinformatics.

## Abstract

**Background:**

Recurrent miscarriage (RM) remains unsolved in > 50% of patients and causes physical and psychological problems in women without specific risk factors for miscarriage. For a successful pregnancy, acceptance of the endometrium and invasion of trophoblast cells into the endometrium is necessary.

**Objective:**

This study aimed to use computational analysis to identify key genes and related pathways in endometrial and trophoblast cells derived from RM samples.

**Materials and Methods:**

In this bioinformatics study, we explored the differential expression of genes in endometrial and trophoblast cells by analyzing the GSE165004 and GSE76862 datasets, respectively with the limma package in R software. Subsequently, overlapped genes between 2 datasets were selected, gene ontology and Kyoto Encyclopedia of Genes and Genomes analyses were performed. The overlapped genes were integrated to construct a protein-protein interaction network and hub genes selection.

**Results:**

We observed 41 overlapped genes between endometrial and trophoblast cells, and future analysis was accomplished in overlapped and nonoverlapped genes. Kyoto Encyclopedia of Genes and Genomes analysis indicated that overlapped genes were significantly enriched in the complement and coagulation cascades, pluripotency of stem cells, and synthesis and degradation of ketone bodies. Gene ontology analysis suggested that the genes were enriched in the cell cycle, apoptosis, and cell division. The top 10 genes included: *IRS1, FGF2, MAPK6, MAPK1, MAPK3, MAPK8, MAPK9, PLK1, PRKACA*, and *PRKCA* were identified from the PPI network.

**Conclusion:**

This study identified the key genes and potential molecular pathways underlying the development of RM. This could provide novel insights to determine the possible mechanisms and interventional strategies associated with miscarriage.

## 1. Introduction

Recurrent miscarriage (RM) is characterized by terminating all identified pregnancies within the uterus (1, 2). The treatment of RM has become a challenging issue in reproductive medicine due to the limited understanding of its pathogenesis. Numerous advances in genetics, immunology, and cell biology have revealed the significant involvement of genes and noncoding RNAs in the onset and progression of RM.

The regulation of cellular processes, including cell cycle, apoptosis, and epithelial-mesenchymal transition, by various transcriptomic networks can impact placental trophoblast growth, migration, and invasion (3). Hence, aberrant expression of various transcriptomic factors may contribute to the onset and progression of recurrent spontaneous miscarriage.

During the primary phases of a typical pregnancy, the proper execution of various functions by the fetal trophoblast significantly impacts the fetus viability (4). Following the successful implantation of the embryo, a process of cellular differentiation arises, whereby the extravillous trophoblast infiltrate the maternal uterine tissue, establishing a fixed attachment of the placenta to the uterus. This event is accompanied by restructuring of the maternal vasculature, which facilitates the provision of vital nutrients to the developing fetus. Any mistake made during this process can result in pathological pregnancies, which may include recurrent spontaneous miscarriages (5, 6). The necessity of cell signaling appears to be evident in facilitating communication between trophoblast and endometrium. Alterations in the concentration of intracellular signaling molecules resulting from genetic modifications and correlated transcripts may give rise to gestational disorders, including RM, pre-eclampsia, and fetal growth restriction (7).

Transcriptome production marks the initial stage of any cell function or structure alteration. Variations in cellular function are attributed to alterations in gene expression. Hence, gene expression analysis can offer a valuable understanding of the disease pathogenesis (8). Transcriptomics investigations have the potential to provide numerous genes as molecular markers (9).

Identifying biomarkers and understanding the complicated process of embryo implantation may facilitate the classification of distinct phases of endometrial acceptance (pre- and post-implantation) and the formulation of tailored pharmacological interventions. Furthermore, the detection of genes that exhibit differential expression genes (DEG) holds significant value in the identification of enrichment pathways, which can aid in the interpretation of molecular processes that have been altered in cases of abortion and the comprehension of anomalous mechanisms of the disease (10).

The present study aimed to examine genes exhibiting alterations in expression within 2 distinct cell groups (endometrium and trophoblast) using analyzing expression data obtained from the gene expression omnibus database. Subsequently, a comparative analysis is conducted between the genetic profiles of both groups, focusing on identifying common and distinct genes.

The following step involves the identification of the biological pathways that are associated with each group of genes.

## 2. Materials and Methods

### Data collection

This bioinformatics study involved retrieving data relating to endometrium and trophoblast from the gene expression omnibus database (http://www.ncbi.nlm.nih.gov/geo/) after initial inquiries. This study investigated 2 different datasets for the problem of recurrent abortion and found an overlap between these 2 datasets. In this approach, better treatment or diagnostic markers can be provided. The dataset concerning the endometrium, identified by the accession code GSE165004, comprises 24 subjects with normal fertility and 24 with RMs. The study of GSE165004 was directed to understand the transcriptomic profile of mid-secretory phase endometria of patients with recurrent pregnancy losses and unexplained infertility by comparing with the endometria of healthy fertile women (controls) by weighted gene co-expression network analysis. However, in our study, only data related to recurrent abortions and healthy women were examined with a different method.

Additionally, the trophoblast data, accessible through the GSE76862 accession code, consists of 3 healthy controls and 3 subjects with RMs. The study included information relating to both asymptomatic women and women with a history of RM, determined by a specialist. The inclusion criteria for the study contained individuals who lacked any abnormalities of uterine and fallopian tubes or obstructions in various uterine segments; moreover, these individuals exhibited a normal karyotype. This study aimed to find the common pathway between 2 important parts in fertility, that is, endometrial and trophoblast cells; however, with the inclusion criteria, there was only one data in the database for each case. Therefore, it was impossible to add more data. Other datasets had different samples or genetic markers.

### DEG analysis in the endometrium and trophoblast

The limma package of R software was employed for analyzing microarray and RNA-seq data. The RMA method for microarray data and counts per million for RNA-seq data were used for normalization and filtering. Initially, an analysis was conducted on the endometrium sample data. Subsequently, the dataset concerning trophoblast was also analyzed following the identification of DEGs. The statistical significance threshold for screening both datasets was determined as adj-p-value < 0.05 and |log ${\;}_{2}$ FC| > 0.5.

Subsequently, the EnhancedVolcano package in the R programming language generated volcano plots representing DEGs. Following the identification of DEGs in each group using a Venn diagram, the overlapped genes between the 2 groups were subsequently determined.

### Enrichment analysis of target genes

The gene ontology (GO) and Kyoto Encyclopedia of Genes and Genomes (KEGG) pathways for both endometrial and trophoblast were determined using the clusterProfiler package and KEGG Orthology-Based Annotation System (KOBAS) online database for common and distinct genes. The cluster profile software package employs the R programming language to analyze GO, which comprises biological processes, molecular functions, and cellular components. The KOBAS database employs machine learning to rank biologically significant pathways effectively. The KOBAS server employs a total of 10 databases for analysis, consisting of 5 pathway databases (KEGG Pathway, PID, BioCyc, Reactome, and Panther) and 5 human databases (OMIM, KEGG Disease, FunDO, GAD, and NHGRI GWAS). To investigate this analysis, both common and distinct genes were uploaded into the database, and enrichment analyses were subsequently conducted.

### Protein-protein interaction (PPI) network and analysis of key genes

PPI concerns the intermolecular interaction between proteins within biological processes. The STRING database, which stands for the retrieval of interacting genes and is accessible via https://string-db.org/, is a web-based server utilized to assess PPI network data. To achieve this objective, the overlapped and distinct genes among the groups were identified, then the network was constructed by utilizing the STRING platform. Subsequently, the corresponding network was generated by Cytoscape software version 3.9.1. The CutoHabba plugin was used within the Cytoscape software platform to determine the hub genes in the network exhibiting the highest score.

### Ethical considerations

This study was ethically approved by the Ethical Committee of Shahrekord University, Shahrekord, Iran (Code: IR.SKU.REC.1402.020).

## 3. Results

### Analysis of differential gene expression data in the endometrium and trophoblast 

Before conducting a differential analysis of gene expression, a quality assessment and normalization of the expression datasets (GSE165004 and GSE76862) were accomplished. To assess data quality, zero values in each sample were surveyed, and subsequently, data relating to zero-valued items were eliminated through filtration. The normalization of data was performed utilizing the techniques provided by the limma package.

Upon analysis of the corrected expression data and utilizing the established significance threshold of p < 0.05 and |log ${\;}_{2}$ FC| > 0.5, 1701 genes were identified as having undergone alterations in expression within the trophoblast dataset. Specifically, 751 genes exhibited increased expression, while 950 genes displayed decreased expression. The study analyzed endometrium data, revealing a total of 1041 genes with differential expressions. Among these genes, 480 exhibited upregulation, while 561 exhibited downregulation.

**Figure 1:**
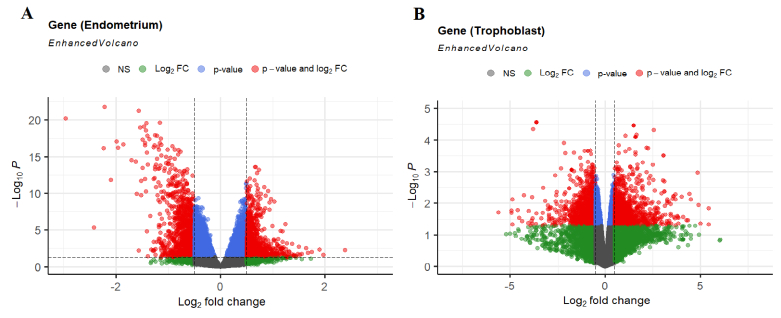
Results of differential analysis shown by volcano plot for A) Endometrium, and B) Trophoblast. Volcano plot showing fold differences in mRNA expression and adjusted p-value relationship for the significance test. Red points represent a dysregulated expression.

**Figure 2:**
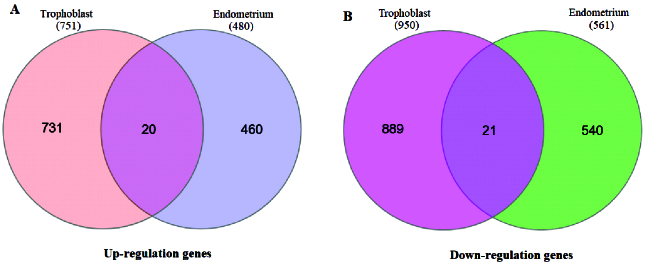
Venn diagram to determine overlapped genes between DEGs with increased expression A) and DEGs with reduced expression B) in the trophoblast and endometrium data.

Tables I and II display the 10 genes with the highest score in up- or downregulation across both datasets. Furthermore, figure 1 shows volcano plots for the analyzed data, with the red data points indicating instances of differential expression. The Venn diagram determined the overlapped genes between 2 distinct groups. Figure 2 reveals that 21 common genes were observed between trophoblast and endometrium DEGs exhibiting decreased expression, while 20 common genes were identified between DEGs exhibiting increased expression.

### Gene enrichment analysis

**Figure 3:**
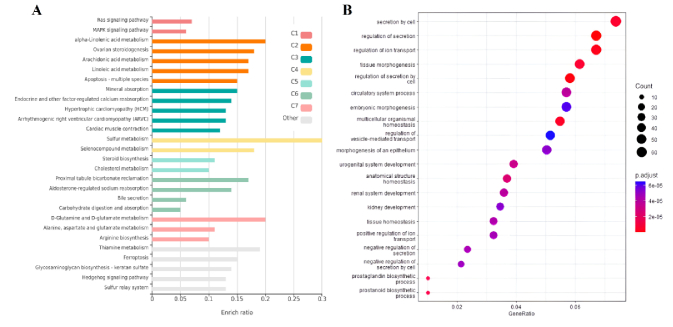
A) KEGG pathways, and B) GO analysis associated with DEGs in the endometrium. The size of the circle is proportional to the count of target genes. The color of the circle defines the adjusted p-value. The 2 ends of the colors are red and blue, depicting lower and higher adj. p-values, respectively. They are corresponding to “secretion by cell” (adj. p-value ≈ 5.98e-06) and “prostanoid biosynthetic process” (adj. p-value ≈ 1.26e-05), respectively.

**Figure 4:**
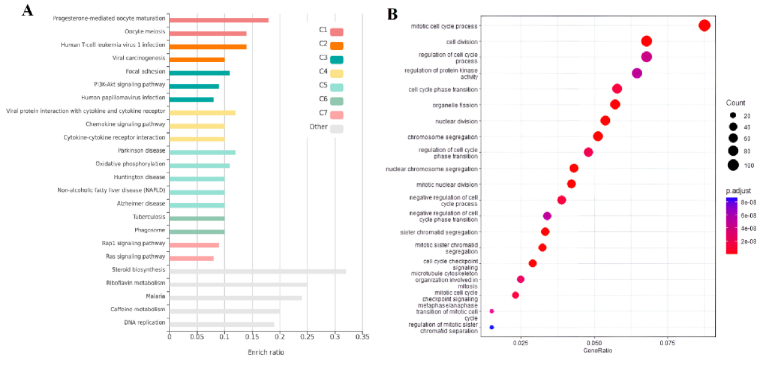
A) KEGG pathways, and B) GO analysis associated with DEGs in trophoblast. The size of the circle is proportional to the count of target genes. The color of the circle defines the adj. p-value. The 2 ends of the colors are red and blue, depicting lower and higher adj. p-values, respectively. They correspond to “mitotic cell cycle process” (adj. p-value ≈ 5.5e-15) and “regulation of mitotic sister chromatid separation” (adj. p-value ≈ 8.7e-08), respectively.

To conduct a more comprehensive examination of overlapped genes between trophoblast and endometrium data, as well as unique genes within each group, GO analysis and analysis of signaling pathways were carried out utilizing the KEGG database. The survey results revealed 20 pathways that obtained the highest score. As per the findings presented in figure 3A, the distinct genes associated with the endometrium, the KEGG database has identified various signaling pathways that include the synthesis and metabolism of compounds such as sulfur, thiamine, cholesterol, linoleic acid, arachidonic acid, and solenoid compounds. Furthermore, apoptosis and cell cycle pathways were most significantly recognized among the pathways. According to the GO analysis conducted on these genes, significant findings were observed in relation to the secretion of cell factors, ion transport, tissue morphogenesis, and embryonic and epithelial cells (Figure 3B). The KEGG pathways analysis revealed significant enrichment of viral infection pathways, including papillomavirus and virus interaction with various receptors, phosphoinositide 3-kinase (PI3K)/protein kinase B (AKT) signaling pathway, cell division, central junctions, and chemokine-dependent signaling in trophoblast (Figure 4A).

**Figure 5:**
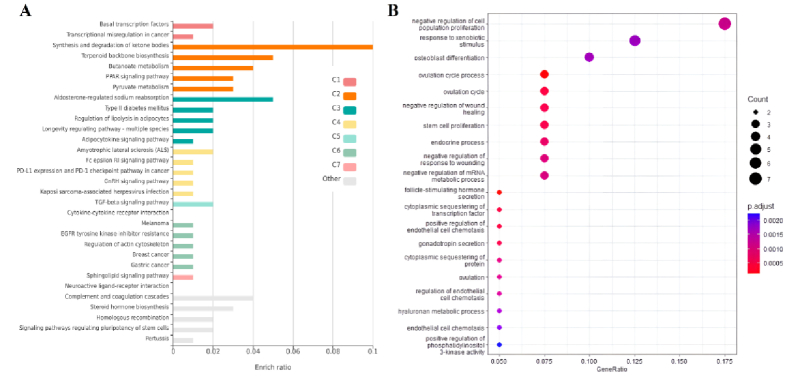
A) KEGG pathways, and B) GO analysis associated with overlapped DEGs between trophoblast and endometrium. The size of the circle is proportional to the count of target genes. The color of the circle defines the adj. p-value. The 2 ends of the colors are red and blue, depicting lower and higher adj. p-values, respectively. They are corresponding to “negative regulation of cell population proliferation” (adj. p-value ≈ 0.0011) and “positive regulation of phosphatidylinositol 3-kinase activity” (adj. p-value ≈ 0.0022), respectively.

The GO analysis in the trophoblast cells revealed a significant enrichment of various items related to cell division, cell cycle, and associated processes, including chromosomal division and separation, as well as the transfer of different phases of the cell cycle (Figure 4B). Upon analyzing common genes, it was observed that a significant number of genes contributed to the biosynthesis and catabolism of ketone bodies, as indicated by the KEGG pathway analysis. Several pathways were identified, including the biosynthesis of terpenoids, pyruvate metabolism, and a cascade related to coagulation and complement (Figure 5A). The study conducted a GO analysis on common genes, which yielded significant findings such as the inhibition of cell proliferation, response to xenobiotic stimuli, and negative regulation of wound healing and stem cell proliferation (Figure 5B).

### PPI network

**Figure 6:**
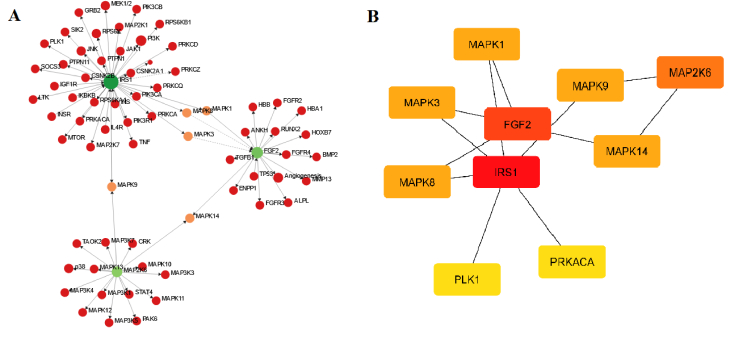
A) Modules identified in the PPI network analysis of common genes based on the most connections and relationships with other proteins. B) Key genes selected in the study were identified by the CytoHabba plugin. The key genes are marked according to the score from the highest score (red color) to the lowest score (yellow color).

The interaction data relating to overlapped genes, and the PPI network were acquired using the STRING database. A network was constructed to represent shared genes, consisting of 74 nodes and 130 edges with a degree of 0.8. The network underwent analysis using Cytoscape software, extracting a key network with the highest score from the STRING network (Figure 6A). The CytoHabba plugin within the Cytoscape software was utilized to identify hub genes. Note that the plugin employs various topological techniques, and in the current study, the degree approach was utilized to identify hub genes. The method used is measured precisely in its identification of crucial proteins, as it relies on the highest degree of connections within the network and subsequently assigns them the highest score (Figure 6B). The key genes identified as significant through analysis in the Cytoscape software were insulin receptor substrate 1 (*IRS1*), fibroblast growth factor 2 (*FGF2*), mitogen-activated protein kinase 6 (*MAPK6*), *MAPK1*, *MAPK3*, *MAPK8*, *MAPK9*, polo like kinase 1 (*PLK1*), protein kinase CAMP-activated catalytic subunit alpha (*PRKACA*), and protein kinase C alpha (*PRKCA*).

**Table 1:** Top 10 downregulated differentially expressed genes in endometrial tissue and trophoblast cells


**Gene**	**adj. P-value **	**Treatment**	**Background**	**Log_2_FC**
*NORAD*	2.19E-35	-32.515397	63.81388	-3.0594497
*TPM4P3*	5.62E-21	-15.677336	36.411673	-2.9637927
*TBCD*	4.57E-28	-22.717048	51.067727	-2.4787172
*KRAS*	4.25E-06	-5.158958	4.03406	-2.419536
*RPL6P2*	4.85E-29	-23.852368	52.265396	-2.4156716
*HBZ*	0.000010	-12.200	-3.175	-7.67
*HBA1*	0.000009	-12.400	-2.767	-6.12
*LEP*	0.019421	-3.080	-4.009	-5.62
*LHB*	0.016681	-3.200	-4.060	-4.94
*CGB8*	0.020182	-3.050	-3.967	-4.93
*NORAD*: Non-coding RNA activated by DNA damage, *TPM4P3*: TPM4 pseudogene 3, *TBCD*: Tubulin folding cofactor D, *KRAS*: Kirsten rat sarcoma virus, *RPL6P2*: Ribosomal protein L6 pseudogene 2, *HBZ*: Hemoglobin subunit zeta, *HBA1*: Hemoglobin subunit alpha 1, *LEP*: Leptin, *LHB*: Luteinizing hormone subunit beta, *CGB8*: Chorionic gonadotropin subunit beta 8				

**Table 2:** Top 10 upregulated differentially expressed genes in endometrial tissue and trophoblast cells


**Gene**	**adj. P-value**	**Treatment**	**Background**	**Log_2_FC**
*C4BPA*	0.001301	5.380	-3.337	8.08
*THBS1*	0.014062	3.320	-3.728	5.44
*CLU*	0.046080	2.460	-3.911	5.42
*CYBB*	0.042781	2.510	-4.007	5.00
*IGFBP7*	0.011479	3.480	-3.762	4.90
*CISD1*	5.32E-03	2.913665	-2.71531	2.3833725
*CRISP3*	2.40E-02	2.327383	-4.057831	1.9717057
*NDRG4*	5.06E-03	2.932151	-2.644794	1.8921431
*FRG1*	9.78E-03	2.686042	-3.165757	1.7494827
*CPT1A*	1.37E-02	2.554778	-3.558119	1.6761157
*C4BPA*: Complement component 4 binding protein alpha, *THBS1*: Thrombospondin 1, *CLU*: Clusterin, *CYBB*: Cytochrome B-245 beta chain, *IGFBP7*: Insulin like growth factor binding protein 7, *CISD1*: CDGSH iron sulfur domain 1, *CRISP3*: Cysteine rich secretory protein 3, *NDRG4*: NDRG family member 4, *FRG1*: FSHD region gene 1, *CPT1A*: Carnitine palmitoyltransferase 1A				

## 4. Discussion

The present study involved selecting and examining 2 distinct sets of samples. The initial cohort comprised endometrium samples, while the subsequent cohort consisted of trophoblasts that were extracted from women who had experienced RMs and healthy controls. Upon conducting a comprehensive analysis of the data, a comparative analysis was performed between the 2 distinct sample types, identifying 41 common genes between the endometrium and trophoblast.

The problem of RMs remains a significant reproductive concern worldwide and a prevalent complication associated with pregnancy.

To understand the abnormality in the fertility and reproductive system leading to fetal loss, it is vital to examine the mechanisms and factors implicated in the ovarian follicle production process and the implantation of the fetus in the maternal uterus, as well as the disorders of these pathways. The processes are greatly influenced by genetics. While the diagnosis of RMs may be challenging, it can be beneficial for both the medical practitioner and the patient, as it enhances the chance of a successful subsequent pregnancy. Thus, identifying effective factors in this regard may be highly advantageous.

Upon studying the pathways implicated in the functioning of overlapped genes, a considerable proportion of genes exhibiting alterations in expression are subject to regulation within the cell cycle pathway. Disruptions in this pathway are associated with the activation of cellular proliferation and the inhibition of apoptosis, as previously reported (11). Moreover, it has been revealed that cell cycle regulation plays a crucial role in the differentiation process of uterine stromal cells during implantation. The genes responsible for regulating the cell cycle exhibit dysregulation during the secretory phase of the endometrium. Hence, regulating gene expression relating to this process through hormone intervention, such as progesterone or steroids, has been observed to be effective in enhancing the implantation rate (12). Also, one of the findings elucidated in this investigation relates to the function of apoptosis mediated by the involved genes. Comparative analyses between samples with miscarriages and healthy samples have revealed a significant increase in the level of apoptosis in the former group. Subsequent investigations have suggested that increased apoptosis may be one of the factors contributing to RMs (13). Apoptosis has been verified to exist in trophoblast during the initial stages of pregnancy. The processes of implantation, blastocyst growth, endometrial regeneration, and placental structure reconstruction, among others, are intricately linked to apoptosis (14).

The balance between cellular proliferation in placental villi and endometrium and the level of apoptosis is crucial in pregnancy physiology. However, in pathological conditions such as miscarriage, this process is disrupted and influenced, leading to an imbalance. The present study displays the PI3K-Akt signaling pathway, which regulates cellular growth and apoptosis. Dysregulation of this pathway increases proliferation or apoptosis within the endometrium (15). Typically, the PI3K-Akt signaling pathway is downregulated in initiating embryo acceptance by the endometrium. During the proliferative phase, the PI3K-Akt signaling pathway expression is diminished in women with a thin endometrium (16).

Thus, the aforementioned approach may also prove effective in abortion. An additional pathway identified is the metabolic pathway of ketone bodies. Ketone bodies are a class of organic compounds characterized by a carbonyl group in the middle of the carbon chain. They are synthesized in the hepatic tissue through the process of ketogenesis. Ketone bodies are a significant substitute fuel for peripheral tissues, particularly in the absence of glucose. Various factors, including glucagon trigger the induction of lipolysis and ketogenesis, while insulin suppresses these processes (17). Research has indicated that pregnant women are at a higher risk of experiencing ketosis than others (18). Numerous investigations, comprising animal model experiments, have demonstrated that environments characterized by elevated ketone levels may result in unfavorable outcomes for both the mother and the fetus (19). The study found that beta-hydroxy-butyric acid exposure in mouse embryos resulted in a range of abnormalities, including growth retardation and neural tube defects. The severity of these abnormalities was found to increase with higher exposure doses (20).

The pathway associated with the activation of the complement system was highly significant among the pathways linked to shared genes. The complement system exhibits activity throughout the menstrual cycle, with heightened expression observed during the secretory phase. The molecules of the complement system impact the attachment between the embryo and the endometrium at the location of implantation. The complement protein C1q has a significant function in the implantation process within the endometrium and is also implicated in the pathogenesis of pre-eclampsia, as evidenced by previous research (21). It has been demonstrated that the coagulation and complement cascade pathway is significant in the endometrium during pregnancy (22). Apart from preserving the endometrium through the preservation of epithelial integrity, the presence of a functional complement system also raises worries regarding the possibility of the fetus being perceived as a foreign object. Thus, it is crucial to establish balance in this case (23).

Furthermore, hub genes were identified in the pathways discovered during the study. *IRS1* and *FGF2* were identified as common genes with the highest score. The *IRS1* gene encodes for the insulin receptor and initiates the insulin-dependent signaling cascade. Insulin is a crucial metabolic hormone in maintaining energy homeostasis within the human body (24). The insulin-dependent signaling pathway is a crucial factor in the development of the fetus. The insulin hormone, acting as a growth factor, can potentially enhance cell proliferation and inhibit apoptosis via the PI3K-Akt pathways in endometrial cancer (25). The invasion behavior of endometrial cancer is associated with the activation of the insulin receptor *IRS1*. Over the initial 20 wk of development, there is a steady increase in fetal growth hormone levels, which significantly impacts insulin metabolism (26). Hence, the balance of energy can impact the embryo implantation process and subsequent growth. The current study demonstrates the reduction in the insulin receptor gene *IRS1* expression among females who experienced miscarriages. The *FGF2* gene is responsible for encoding the fibroblast growth factor- biomolecules that induce cellular growth, proliferation, and homeostatic regulation. These factors are crucial in regulating proliferation, differentiation, angiogenesis, tissue regeneration, and embryo development (27).

The expression of *FGF2* is observed to be elevated in the endometrium during the gestational period. The literature indicates that *FGF2* has demonstrated the ability to induce trophoblast migration, adhesion, and embryogenesis across multiple species (28). In addition, it has been established that *FGF2*, in conjunction with vascular endothelial growth factor, plays a crucial role in regulating the angiogenesis process during placental development (29). The observed reduction in *FGF2* expression in samples associated with RMs highlights this factor's significance in maintaining fetal viability throughout gestation.

## 5. Conclusion

The current study aimed to employ bioinformatics methodologies and tools to identify significant genes and their associated pathways in endometrium and trophoblasts. Furthermore, a comparative analysis was conducted to identify the common genes between the 2 groups by examining the genes exhibiting alterations in expression across both tissues. The study's findings indicate that the genes analyzed in both endometrial and trophoblastic samples are significant in women with RMs compared to those with normal pregnancies. Conversely, the co-occurrence of common genes in the aforementioned groups simultaneously underscores the significance of accuracy in both cell types.

##  Data availability

The data generated during and/or analyses during the current study are available from the corresponding author upon reasonable request.

##  Author contributions 

K. Ahmadi performed most of the bioinformatics data analysis. S. Reiisi coordinated the study, participated in intellectual discussions of the data, and wrote and revised the manuscript. Z. Habibi participated in intellectual discussions of the data and helped in reviewing the manuscript. All authors approved the final manuscript and take responsibility for the integrity of the data.

##  Conflict of Interest

The authors declare that they have no conflict of interest.
